# An exploratory study of recognition behaviors practiced by mid-level nurse managers and perceived by mid-level staff nurses

**DOI:** 10.20407/fmj.2025-023

**Published:** 2026-02-28

**Authors:** Yuya Saito, Hiroe Koyanagi, Keiko Mano, Ryoko Murayama

**Affiliations:** 1 Graduate School of Health Sciences, Major in Medical Sciences, Fujita Health University, Toyoake, Aichi, Japan; 2 Division of Nursing, Fujita Health University Hospital, Toyoake, Aichi, Japan; 3 Faculty of Nursing, School of Health Sciences, Fujita Health University, Toyoake, Aichi, Japan; 4 Research Center for Implementation Nursing Science Initiative, Fujita Health University, Toyoake, Aichi, Japan

**Keywords:** Recognition behavior, Mid-level nurse managers, Mid-level staff nurses, Work engagement, Qualitative research

## Abstract

**Objectives:**

This study explored mid-level nurse managers’ recognition behaviors and the perception of those behaviors by mid-level staff nurses. This information is needed for nurse manager training, and may improve nurses’ work engagement and retention in the workforce.

**Methods:**

The exploratory qualitative study gathered data through semi-structured interviews with mid-level nurse managers and nurses selected through purposive sampling at a large acute care hospital in Aichi, Japan. Data were analyzed using Bryman’s content analysis, and categories were generated both deductively and inductively. The *Recognition Behavior Scale of Nurse Managers* was used for deductive analysis. Inductive analysis enabled emergence of new themes.

**Results:**

Five recognition behavior frameworks were identified from 91 (nurse managers) and 50 (staff nurses) codes: *Close Communication*, *Supportive Consultation/Advice*, *Comfortable Attention*, *Positive Performance Evaluation*, and a newly identified framework, *Recognition Environment Fostering*. Mid-level nurse managers practiced behaviors such as ensuring recognition from appropriate individuals and considering nurses’ family situations. Staff nurses emphasized the importance of involvement in ward operations, receiving recognition from managers, and being entrusted with mentoring roles. Certain recognition behaviors were common across both groups. However, some managers expressed uncertainty regarding the effectiveness of their recognition practices.

**Conclusion:**

This study identified key recognition behaviors commonly understood by mid-level nurse managers and staff nurses. These shared behaviors could serve as foundational elements for developing educational programs that support mid-level nurse managers in practicing recognition behaviors more consciously and effectively. Such programs may contribute to fostering work engagement and improving retention of mid-level staff nurses.

## Introduction

Japan’s shortage of nursing staff presents a serious workforce problem for the country.^1^ In particular, insufficient numbers of mid-level staff nurses are reported.^2^ Mid-level staff nurses are typically in a critical period of career development and in their late 20s–30s. They are vital members of the healthcare team who lead advanced nursing practice and contribute significantly to patient satisfaction.^3^ Given their essential role in maintaining the quality of nursing, the resignation of mid-level staff nurses from hospitals is a critical issue.

Support that focuses on stress management is important to promote retention among mid-level staff nurses.^4^ Additionally, retention strategies that reduce stress and promote the engagement of individuals and organizations have been increasingly emphasized.^5^ Work engagement is a pertinent concept correlated with health, organizational commitment, intention to leave, and performance in previous meta-analyses.^6^ It is defined as “a positive, fulfilling, work-related state of mind” characterized by commitment and absorption.^6^ High levels of work engagement may also positively impact others.^7^ Work engagement is enhanced by personal resources (e.g., internal characteristics like self-efficacy, optimism, and self-esteem) and job resources (e.g., tasks performed at work and support from supervisors). Thus, support that enriches both personal and job resources among staff can help foster work engagement.^8^ The Job Demands–Resources model illustrates the relationship between work engagement and job satisfaction,^9^ defined as a positive perception of one’s work.

In healthcare, emotional and relational skills such as communication and respect are as important as technical skills. Job satisfaction among nurses is associated with fair compensation, meaningful recognition, and transparent evaluation systems.^10^ Communication by nurse managers and respectful actions significantly contribute to job satisfaction.^11^ Nursing manager leadership – behaviors or actions that recognize nurses’ responsibilities – is the strongest factor for improving workplace engagement.^12^ Although monetary rewards are often considered a form of recognition,^13^ financial incentives are typically beyond the authority of nurse managers. The International Labour Organization (Recommendation no. 157) stipulates that compensation for nurses should be determined through collective bargaining.^14^ Therefore, nurse managers should focus on culturally appropriate and context-sensitive recognition behaviors that emphasize non-monetary forms of support.

In Japan, studies indicate the need to investigate the influence of organizational efforts by nurse managers on the work engagement of their staff. One study highlighted recognition from supervisors as a key resource for nurturing work engagement among nurses.^15^ Hagimoto et al. developed the *Recognition Behavior Scale of Nurse Managers*^16^ to evaluate recognition behaviors that provide emotional support for nurses in the cultural context of Japan. Thus, properly practicing recognition behaviors is a critical aspect of nursing management.^17^ However, programs focusing on recognition from supervisors as a means to enhance work engagement, particularly those led by nurse managers, are yet to be developed. Accordingly, this exploratory study aimed to identify the recognition behaviors practiced by mid-level nurse managers and perceived by mid-level staff nurses in clinical settings. This information could contribute to the development of educational programs for nurse managers, training them to practice effective recognition behaviors. Ultimately, such efforts may improve work engagement among mid-level staff nurses.

## Methods

### Study design

This study employed a qualitative exploratory research design, which was appropriate for a study contributing to the development of an educational program. Data collection took place through semi-structured interviews.

### Participants

The participants were mid-level nurse managers and nurses working at a large acute care hospital in Aichi, Japan. Participants were recruited through purposive sampling, and mid-level nurse managers were selected following recommendation from their department heads, who identified them as individuals consciously practicing recognition behaviors. Mid-level nursing managers were selected from head nurses responsible for a nursing unit in an acute care hospital, excluding those without experience managing a unit with subordinates and those with less than one year of managerial experience. Mid-level staff nurses were selected from nurses without managerial positions who were Level III or higher on the Japanese Nursing Association’s Clinical Ladder, excluding those with less than one year of employment at their current hospital. The researchers obtained informed consent from the recommended participants. To minimize potential interpersonal influence and ensure independent perspectives, mid-level nurse managers and mid-level staff nurses were not recruited from the same nursing units.

### Data collection

The research site was a single acute care hospital with more than 1,000 beds. Data were collected from March 8 to May 31, 2024. Semi-structured interviews were conducted using an interview guide developed by the research team that aimed to elicit concrete recognition-related experiences. For mid-level nurse managers, questions included prompts such as describing episodes in which they felt recognized by their supervisors when working as staff nurses, and recounting success or failure in their own recognition behaviors as managers. Mid-level staff nurses were asked to reflect on their career and relationships with nurse managers, and to describe experiences of being recognized, praised, or encouraged by their supervisors. With participants’ permission, interviews were audio-recorded using a digital IC recorder to ensure the accuracy of transcription and analysis.

The interview guide was refined through a pilot interview with a mid-level nurse manager experienced in qualitative interviewing, enhancing the appropriateness and depth of the questions. Basic demographic data were also collected, including gender, years of nursing and managerial experience, history of completion of the Certified Nurse Manager Training Programs, and number of subordinates.

### Ethical considerations

The study adhered to ethical guidelines based on the Declaration of Helsinki.^18^ Ethical approval was obtained from the Fujita Health University Health Research Ethics Committee (HM23-370). Informed consent was obtained from all participants. All interviews were conducted in private rooms and limited to a maximum duration of 90 minutes. Personal identifiers in the data were replaced with codes to ensure anonymity.

### Data analysis

Verbatim transcripts were created from the audio recordings. These transcripts were thoroughly reviewed by the research team, and segments that represented recognition behaviors were extracted. Recognition behavior was defined as actions (verbal or nonverbal) that explicitly demonstrated acknowledgment or appreciation toward nurses. These behaviors were then labeled with codes reflecting their characteristics. The coding process was conducted independently by the first author and verified through discussion with co-authors who possess substantial expertise in qualitative research, thereby ensuring consistency and analytical rigor. The narratives regarding recognition behaviors were analyzed using Bryman’s qualitative content analysis method.^19^ Deductive and inductive approaches were used to ensure a comprehensive understanding of the data. Categories were generated deductively based on the four factors of the Recognition Behavior Scale of Nurse Managers,^16^ and inductively to allow for the emergence of new themes.

### Study rigor

This study employed the Standards for Reporting Qualitative Research to ensure credibility, dependability, and transferability.^20^ Although the primary interviewer had limited experience in nursing management, the data were analyzed in close collaboration with the co-authors who had extensive managerial experience. Sampling continued until data saturation was reached. Verbatim transcripts were generated from audio recordings, and the data were analyzed through deductive and inductive approaches. All authors engaged in discussions to ensure interpretive validity and analytical trustworthiness. These measures collectively contribute to the trustworthiness of the findings.

## Results

### Participant characteristics

The mid-level nurse managers included five women and three men. All eight had completed the First-Level Certified Nurse Manager Training Program, and five (62.5%) had completed the Second-Level Program. On average, the mid-level nurse managers had 22 years of nursing experience, 7 years of managerial experience, and 36 subordinates. Conversely, the mid-level staff nurses included six women and one man, with a mean of 12 years of nursing experience (Table 1).

### 1. Qualitative content analysis

Five frameworks of recognition behaviors were identified via qualitative content analysis: *Close Communication*, *Supportive Consultation/Advice*, *Comfortable Attention*, *Positive Performance Evaluation*, and *Recognition Environment Fostering*. The first four frameworks are based on the four factors of the *Recognition Behavior Scale of Nurse Managers*,^16^ whereas the fifth one (*Recognition Environment Fostering*) is a new framework that emerged inductively during the analysis. Verbatim quotations from participants are used to illustrate categories in the sections that follow.

#### 1-1. Recognition behaviors practiced by mid-level nurse managers

A total of 91 codes related to recognition behaviors were extracted from the interviews with nurse managers and subjected to qualitative content analysis (Table 2).

a. *Close Communication*. From the 22 codes, four categories were generated.

Demonstrating Deep Personal Interest: In addition to concern for the nurse’s physical and mental health, this behavior includes consideration for their family members.

*“I always make sure to check in when a staff member has a sick family member.”*

This subcategory, which highlights attention to nurses’ family lives, was not part of the original Recognition Behavior Scale, and was therefore a novel finding.

Responding with Immediacy: This behavior involves responding immediately to troubles or concerns while maintaining a listening stance.

*“If a staff member talks to me, I always stop what I’m doing and listen.”*

This subcategory emphasizes the immediacy of managerial response and was included as a new element not captured in the existing scale.

Conveying the Manager’s Intentions: Managers clearly emphasized supporting the personal and professional growth of their staff, prioritizing this over departmental or institutional profitability.

*“I want my staff to grow professionally. That matters more to me than profits for the ward or hospital.”*

Implementing Ward Management that Reflects Individual Opinions: Instead of issuing unilateral directives, managers engaged staff in decision-making processes, thereby fostering a sense of ownership and collaboration.

*“When initiating a new project, I always seek input from staff rather than making decisions alone.”*

b. *Supportive Consultation/Advice*. From 16 codes, four categories were generated.

Communicating Achievements to Others: Individual accomplishments are recognized and shared with the team.

*“When a staff member achieves something that others struggle with, I intentionally share their success at the nurses’ station.”*

Providing Individual Feedback: Personal, verbal feedback on performance is given.

*“At the end of a shift, I think it makes a difference whether I say ‘Great job today’ or not.”*

Providing Timely Educational Support: Direct guidance is offered in real time.

*“Let’s look it up together. We have a tablet, so let’s check and resolve it on the spot.”*

Providing Guidance with Consideration for Context: This behavior involved thoughtful attention to the timing and setting in which feedback – positive and negative – was delivered. Managers deliberately avoided offering criticism in public and ensured that praise was given in a manner appropriate to the situation.

*“I refrain from criticizing staff in front of others and make sure to offer praise at a time and place that feels appropriate.”*

c. *Comfortable Attention*. Three categories were generated from 14 codes.

Creating Opportunities for Daily Conversations: This behavior involves initiating greetings and spending time with the staff.

*“I make sure to greet everyone every day.”*

Show that you are Watching your Staff: Managers demonstrate to the staff that they are being closely monitored, e.g., noticing changes in their appearance and personal progress.

*“Did you change your hairstyle? Have you lost weight?”*

Expressing Manager’s Positive Emotions: This behavior involves displaying sincere gratitude.

*“I smile and say thank you from the heart.”*

d. *Positive Performance Evaluation*. From 16 codes, three categories were generated.

Demonstrating Expectations for Individual Abilities: Tasks are assigned on the basis of the individual competencies of the staff.

*“Among my peers, everyone has different strengths, so I carefully assign roles accordingly.”*

The act of acknowledging nurses’ creative ideas and approaches was not originally featured in Hagimoto’s scale and is a new finding presented here.

Evaluating the Process Leading to Achievements: This behavior involves recognizing the steps taken toward achieving results.

*“Rather than just assigning tasks, I help them discover the kind of nursing they want to practice.”*

Providing Compensation for Competence: This reflected the manager’s commitment to formally recognizing individual abilities and achievements through career advancement opportunities. Competent staff members were recommended for leadership roles as a form of acknowledgment and reward.

*“I make sure to recognize staff members’ competence by recommending them for leadership positions.”*

e. *Recognition Environment Fostering*. Four categories were generated from 23 codes.

Receiving Recognition from the Most Suitable Personnel: Recognition practices are assigned to suitable staff who excel in this area.

*“I let someone who excels at recognition take charge of motivating the staff.”*

Conveying Recognition from Others to the Individual: Individuals are informed whenever their colleagues give them recognition.

*“A junior nurse said they wanted to be like you.”*

Efficient Collection of Recognition Information: This behavior involves collaborating with other managers to gather insights about staff performance.

*“Unless we observe the staff’s behavior and nursing practice on a regular basis, we won’t be able to notice how much they’ve grown.”*

Understanding the Traits of Recognizers: Personal tendencies that can affect recognition practices are acknowledged.

*“I’m not good at giving praise.” “There are personality differences between staff members.”*

Some mid-level nurse managers demonstrated efforts to foster a recognition-friendly environment by adjusting their leadership styles to fit individual staff characteristics, and by deliberately creating opportunities for others to express appreciation. These behaviors reflect a systemic approach to promote a culture where recognition is a natural part of daily interactions.

#### 1-2. Ambivalence and uncertainty in practicing recognition behaviors

Some mid-level nurse managers expressed uncertainty or internal conflict regarding their recognition behaviors. They stated: *“I want to know how other managers practice recognition,”* and *“I never feel like I did it right – it’s always hard to tell if it was effective.”* These comments reflect the psychological burden and ambiguity some managers experience when trying to acknowledge staff effectively.

### 2. Recognition behaviors perceived by mid-level staff nurses

A total of 50 codes were extracted from the interviews with staff nurses and subjected to qualitative content analysis (Table 3).

a. *Close Communication*. From 16 codes, four categories were generated.

Demonstrating Deep Personal Interest: This behavior involves being considerate of the nurse’s family context.

*“My manager was considerate when my child was sick or when my husband’s work schedule was difficult.”*

Recognition that includes consideration for the nurse’s family represents a novel dimension not addressed in prior scales.

Demonstrating a Sincere Attitude: Nurse managers exhibited sincerity by promptly addressing staff concerns and maintaining a focused, respectful presence during interactions. This included stopping their current tasks and engaging in direct, eye-level communication.

*“When consulted, the manager stops what they are doing and maintains eye-level contact, demonstrating full attentiveness.”*

Conveying the Manager’s Intentions: Rather than providing superficial or formulaic responses, managers articulated their genuine perspectives as leaders. This approach emphasized honest dialogue and built trust among staff.

*“The nurse manager expressed their candid thoughts as a supervisor rather than offering quick, impersonal responses.”*

Listening to Individual Opinions: The opinions of the staff are heard and considered when making management decisions.

*“The manager always prioritizes listening to frontline opinions.”*

b. *Supportive Consultation/Advice*. From the ten codes, three categories were generated.

Continued Learning: Educational support is given to facilitate a culture of continuous learning.

*“Even though I know I should be guiding junior nurses, I still want to keep learning myself.”*

Support provided during career transitions was identified as a new recognition behavior and is reported as a unique finding in this study.

Providing Individual Feedback: Direct verbal feedback on clinical practice is given.

*“Words from my manager stick with me.”*

Providing Career Development Support: Support is given regarding career advancement and future opportunities.

*“My manager took my career goals seriously and researched opportunities for me.”*

c. *Comfortable Attention*. Two categories were generated from the 10 codes.

Ensuring Equal Communication: All staff members are treated equally.

*“I see my manager greeting every staff member each morning.”*

Show that you are Watching your Staff: This behavior involves observing, understanding, and responding to nonverbal cues.

*“As a nurse, I want my managers to notice nonverbal expressions.”*

d. *Positive Performance Evaluation*. From nine codes, three categories were generated.

Evaluating Daily Nursing Work: In addition to recognizing achievements, good nursing practices and skills are acknowledged.

*“My manager encouraged me to pass on my personal experiences and nursing skills to juniors.”*

Demonstrating Expectations for Individual Abilities: The staff are assigned roles as educators or mentors.

*“My manager told me, ‘I trust you to mentor the juniors’.”*

Providing Compensation for Competence: This behavior highlighted the recognition of individual skills and accomplishments through tangible rewards such as promotions. Staff members who demonstrated competence were formally recommended for advancement.

*“The staff member was recommended for a managerial position in recognition of their demonstrated competence.”*

e. *Recognition Environment Fostering*. From five codes, two categories were generated.

Fostering a Sense of Belonging in Hospital Operations: Involvement in ward operations is encouraged.

*“They created an atmosphere where I could share my knowledge.”*

Conveying Recognition from Others to the Individual: Positive feedback from others is relayed to the individual.

*“My manager said, ‘Your juniors trust you’.”*

Mid-level staff nurses recognized the value of a work environment where recognition was not limited to one-on-one interactions with managers, but was facilitated through team culture and peer involvement. Moreover, they appreciated when managers created a positive atmosphere that encouraged mutual recognition among staff.

## Discussion

Five frameworks of recognition behaviors practiced by mid-level nurse managers and mid-level staff nurses were identified: a) *Close Communication*, b) *Supportive Consultation/Advice*, c) *Comfortable Attention*, d) *Positive Performance Evaluation*, and e) *Recognition Environment Fostering*. The first four frameworks were consistent with the factors outlined in the *Recognition Behavior Scale of Nurse Managers*,^16^ while the fifth was determined inductively, thus providing a new dimension of recognition behavior. Some behaviors practiced by nurse managers were aligned with those recognized by staff nurses, highlighting their shared perceptions and areas for improvement. These findings provide a valuable foundation for developing an educational program for mid-level nurse managers to implement effective recognition behaviors in clinical settings.

### Recognition behaviors practiced by mid-level nurse managers

a. *Close Communication*

One notable behavior identified in this category was demonstrating consideration for nurses’ families – behavior not currently included in the *Recognition Behavior Scale of Nurse Managers*. Such behaviors indicate that managers holistically consider nurses’ environments by focusing not only on the nurses themselves but also on their families (i.e., spouses or children). Nurse managers play a role in supporting the career development of mid-level staff nurses.^21^ These behaviors reflect a recognition of individual values and lifestyles given that these nurses are often in the midst of life events such as marriage or child-rearing.

b. *Supportive Consultation/Advice*

This category includes behaviors such as communicating achievements to others, in line with the *Recognition Behavior Scale of Nurse Managers*.^22^ Nurses are often hesitant to receive or display recognition in public settings. However, mid-level staff nurses may benefit from public acknowledgment because they tend to seek internal validation and evidence of commitment from their managers.

c. *Comfortable Attention*

This category highlights the importance of spending time with staff. Studies show that staff who find it difficult to communicate tend to have less interaction with supervisors, and thus managers are expected to proactively create a communicative environment.^23^ The mid-level nurse managers in this study intentionally shared break times with their staff to create opportunities for recognition behaviors. Behaviors such as noticing changes in appearance reflect an effort to initiate communication through subtle observations.

d. *Positive Performance Evaluation*

The notable behaviors in this category involved evaluating the processes that lead to performance and expressing expectations regarding individual abilities. Previous studies have indicated how nurse managers contribute to the growth of mid-level staff nurses.^24^ Evaluating and assigning roles based on individual capabilities can promote self-reflection and development among nurses.

e. *Recognition Environment Fostering* (Newly Identified Framework)

New behaviors were identified within this framework, such as receiving recognition from the most suitable personnel, conveying recognition from others, efficient collection of recognition information, and understanding the traits of recognizers. These behaviors, which support the recognition given by others, enhance the self-efficacy and autonomy of mid-level staff nurses. Previous studies regarding the recognition received from various sources found that managers, along with patients, families, and physicians, all play a role in enhancing job satisfaction.^25^ Collaborating with other managerial staff in gathering information may be a deliberate strategy to ensure that recognition is delivered by the most appropriate individuals.

Behaviors that showed concern for the individual by attempting to understand them were common across all categories. Such tendencies have previously been identified as behavioral characteristics linked to the recognition behaviors of nurse managers.^26^ These behaviors highlight the importance of attentiveness and sensitivity among individuals and groups, in the context of recognition efforts.

### Recognition behaviors perceived by mid-level staff nurses

a. *Close Communication*

Respect and consideration for nurses’ life plans and work-life balance, including their families, were noted as recognition behaviors by mid-level staff nurses. Supporting work-life balance also helps strengthen family functioning.^27^ Thus, receiving such forms of support from nurse managers may enhance nurses’ personal and professional lives.

b. *Supportive Consultation/Advice*

Staff nurses were given guidance and support in the context of education/learning. The self-directed learning capabilities of mid-level staff nurses have been noted in previous studies, highlighting the importance of experiential learning in career development.^28^ The nurses in this study recognized their roles as educators while also experiencing anxiety about their teaching abilities, indicating their desire for continued learning and support.

c. *Comfortable Attention*

This category highlighted behaviors such as conversing with staff regarding daily observations. Some nurses regarded professional behavior as a given and expressed expectations for their supervisors to notice their nonverbal efforts, such as subtle, unspoken cues in their daily actions. Previous studies describe how mid-level staff nurses perceive and are influenced by recognition from others.^29^ Recognition is an important aspect for supporting nurses in their ongoing struggle to fulfill their roles and responsibilities.

d. *Positive Performance Evaluation*

This category included behaviors that reinforce expectations for individual abilities by assigning mentoring/teaching roles to mid-level staff nurses. In line with this, studies have found that expectations placed on nurses by their colleagues and managers are perceived as a form of recognition.^29^ These expectations improved the quality of nursing practice and created a sense of meaningful engagement through mentorship.

e. *Recognition Environment Fostering* (Newly Identified Framework)

Behaviors such as fostering a sense of belonging and conveying recognition from others were identified within this new framework. Mid-level staff nurses felt that these behaviors were vital contributors to ward operations and promoted an open and communicative environment. In a previous study that developed a leadership scale for mid-level staff nurses, elements such as assertive communication, junior-focused support, and cooperative self-management were considered core competencies.^30^ Thus, mid-level staff nurses should receive recognition but also contribute to a culture of mutual recognition within the organization. By understanding and cooperating with the recognition behaviors practiced by their supervisors, nurses can help create a positive and effective environment of recognition.

### Shared perspectives between mid-level nurse managers and staff nurses

Mid-level nurse managers and nurses recognized the importance of building an environment conducive to recognition behaviors. This gave rise to the new framework: *Recognition Environment Fostering*. Notably, participants varied in their years of nursing and managerial experience, and thus the extracted categories were not limited by experience level.

In the *Close Communication* category, similar behaviors were described by both groups. Most nurses are female, and it is common for them to leave the workforce for family responsibilities. Thus, behaviors that reflect consideration for family members (e.g., children or aging parents) may be perceived as organizational efforts to support working mothers. This recognition behavior is particularly important in female-dominated professions, such as nursing.

Many of the recognition behaviors identified in this study overlap with those in the *Recognition Behavior Scale of Nurse Managers*. However, the original scale was developed based on input from nurses across all age groups and roles, whereas this study focused specifically on mid-level staff nurses, representing a more targeted demographic.

Although there were common items across both groups, the study did not pair managers and nurses from the same units. Therefore, it cannot be concluded that recognition behaviors practiced by managers were directly perceived as such by the nurses. Nevertheless, these identified behaviors offer valuable insights for designing educational programs for mid-level nurse managers, especially considering the similarities between the groups.

### Bridging perceptual gaps and enhancing confidence through recognition behavior education

This study provided two critical insights relevant to the development of recognition behavior education for mid-level nurse managers. First, across the five frameworks, certain recognition behaviors were perceived only by mid-level staff nurses and not by mid-level nurse managers. These differences may reflect unconscious behaviors or gaps in recognition awareness, emphasizing the importance of bridging perceptual gaps between managers and staff. Making nurse managers aware of behaviors that staff value (but which often go unrecognized) could enhance the precision and effectiveness of educational interventions.

Second, some managers expressed a lack of confidence in their recognition behaviors and reported experiencing internal conflict regarding the effectiveness and appropriateness of their practices. Previous studies have indicated that experience with nursing management is necessary for managers to appropriately practice recognition behaviors.^31^ The behaviors identified in this study may serve as reflective benchmarks to help managers evaluate and refine their own recognition behaviors. These findings underscore the necessity of structured educational programs that impart recognition behavior skills and enhance nurse managers’ confidence and clarity in applying them effectively.

Although some categories in this study had very few codes, the findings were interpreted from the perspective of “concrete universality,” wherein deep insights from individual experiences can offer universally applicable meanings. These specific experiences may yield transferable insights applicable to diverse clinical settings and nursing populations.

### Limitations

The small number of participants meant that data collection was limited to a specific facility and environment. Thus, the recognition behaviors of mid-level nurse managers and mid-level staff nurses in other regions or nursing environments may not have been fully captured or represented.

## Conclusion

This study identified recognition behaviors practiced by mid-level nurse managers and perceived by mid-level staff nurses. Several behaviors overlapped between the two groups, suggesting a shared understanding of core recognition elements such as communication, feedback, and performance evaluation. A new framework, *Recognition Environment Fostering*, was identified, highlighting the importance of environmental and systemic support in facilitating effective recognition.

The findings suggest the need for structured educational programs to enhance mid-level nurse managers’ recognition competencies. Such programs may support managers in confidently and consistently practicing recognition behaviors, ultimately contributing to improved work engagement and retention of mid-level staff nurses.

## Figures and Tables

**Table 1:** Participant characteristics

Characteristics		Mid-level nurse managers (*n*=8)				Mid-level nurses (*n*=7)		
		*n* (%)	Mean	Median (range)^a^		*n* (%)	Mean	Median (range)^a^
Gender	Men	3 (37.5)				1 (14.3)		
	Women	5 (62.5)				6 (85.7)		
Years of experience			22	23 (16–29)			12	10 (6–23)
Interview duration (min)			44	41 (37–57)			26	27 (19–31)
Years of nursing management experience			7	6.5 (2–18)				
Number of subordinates			36	33 (24–55)				
Certified nursing manager education^b^	First-level course	8 (100)						
	Second-level course	5 (62.5)						

^a^ Values are expressed as median (range). ^b^ The Certified Nursing Manager Education Course, administered by the Japanese Nursing Association, includes first-, second-, and third-level programs designed to develop managerial competencies required for qualification as a Nurse Manager in Japan.

**Table 2:** Recognition behaviors practiced by mid-level nurse managers

Framework^a^	Category	Subcategory	Code Count
(a) Close Communication	Demonstrating Deep Personal Interest	Showing concern for nurses’ physical and mental well-being	4
		Recalling previous conversations	3
		Considering family circumstances	3
	Responding with Immediacy	Always available to listen	4
		Responding promptly to issues	1
	Conveying the Manager’s Intentions	Communicating honest opinions	2
	Implementing Ward Management that Reflects Individual Opinions	Gathering opinions	2
		Clarifying leadership role	1
		Using visual aids for explanation	1
		Delegating specific responsibilities	1
(b) Supportive Consultation/Advice	Communicating Achievements to Others	Acknowledging and sharing staff achievements	6
	Providing Individual Feedback	Delivering performance-specific comments	4
	Providing Timely Educational Support	Offering real-time educational input	4
	Providing Guidance with Consideration for Context	Ensuring appropriate time/place for feedback	2
(c) Comfortable Attention	Creating Opportunities for Daily Conversations	Initiating greetings and casual interactions	2
		Spending time with staff	2
		Valuing daily exchanges	1
	Show that you are Watching your Staff	Noticing staff appearance	2
		Monitoring progress	2
		Demonstrating sustained attentiveness	1
		Avoiding hierarchical barriers	1
	Expressing Manager’s Positive Emotions	Smiling and expressing gratitude	1
		Showing authentic emotional reactions	1
		Emphasizing words of appreciation	1
(d) Positive Performance Evaluation	Demonstrating Expectations for Individual Abilities	Discussing goals and developmental plans	5
		Recognizing creativity	3
		Assigning meaningful roles	2
		Involving in mentoring tasks	1
	Evaluating the Process Leading to Achievements	Using verbal and visual feedback	2
		Focusing on efforts, not just outcomes	2
	Providing Compensation for Competence	Recommending salary or promotion	1
(e) Recognition Environment Fostering (Newly Identified Factor)	Receiving Recognition from the Most Suitable Personnel	Recognition from relevant individuals	7
	Conveying Recognition from Others to the Individual	Transmitting praise from external sources	7
	Efficient Collection of Recognition Information	Collecting staff data for recognition	3
		Understanding individual strengths	2
		Engaging senior nurses in recognition	2
	Understanding the Traits of Recognizers	Considering the personality of evaluators	2

^a^ Based on the four-factor model of the Recognition Behavior Scale of Nurse Managers (Hagimoto et al., 2014), with one additional framework developed inductively.

**Table 3:** Recognition behaviors perceived by mid-level staff nurses

Framework^a^	Category	Subcategory	Code Count
(a) Close Communication	Demonstrating Deep Personal Interest	Caring for physical and mental well-being	2
		Respecting life decisions	1
		Promoting work-life balance	1
		Expressing concern for the nurse’s family	1
	Demonstrating a Sincere Attitude	Actively listening and reviewing documents	3
		Carefully checking submitted reports	1
	Conveying the Manager’s Intentions	Transmitting motivation	1
		Expressing the manager’s honest thoughts	1
	Listening to Individual Opinions	Respecting and applying on-site feedback	2
		Avoiding dismissive responses	2
		Assigning suitable responsibilities	1
(b) Supportive Consultation/Advice	Continued Learning	Collaborating and offering advice	3
		Supporting career transitions	2
	Providing Individual Feedback	Verbal evaluation of performance	3
	Providing Career Development Support	Sharing information and opportunities	2
(c) Comfortable Attention	Ensuring Equal Communication	Interacting with all staff equally	4
		Maintaining egalitarian attitudes	2
	Show that you are Watching your Staff	Monitoring behavior and engaging staff	4
(d) Positive Performance Evaluation	Evaluating Daily Nursing Work	Acknowledging work performance	1
		Complimenting routine nursing practices	1
		Recognizing efforts to prevent accidents in advance	1
		Praising written documents created by staff	1
	Demonstrating Expectations for Individual Abilities	Assigning roles to experienced nurses	3
		Entrusting junior nurse training	1
	Providing Compensation for Competence	Recommending salary/promotion	1
(e) Recognition Environment Fostering (Newly Identified Factor)	Fostering a Sense of Belonging in Hospital Operations	Encouraging open communication	1
		Listening to team dynamics	1
		Creating a conducive atmosphere	1
	Conveying Recognition from Others to the Individual	Communicating respect and trust	2

^a^ Based on the four-factor model of the Recognition Behavior Scale of Nurse Managers (Hagimoto et al., 2014), with one additional framework developed inductively.
